# Effects of Cerebellar Transcranial Direct Current Stimulation on Cerebellar Brain Inhibition as a Function of TMS Coil Orientation

**DOI:** 10.1002/brb3.70364

**Published:** 2025-02-19

**Authors:** Katharina M. Steiner, Seyed Ali Nicksirat, Andreas Thieme, Lara Müntefering, Thomas M. Ernst, Dagmar Timmann, Giorgi Batsikadze

**Affiliations:** ^1^ Department of Neurology and Center for Translational Neuro and Behavioral Sciences (C‐TNBS), Essen University Hospital University of Duisburg‐Essen Essen Germany; ^2^ LVR‐University‐Hospital Essen, Department of Psychiatry and Psychotherapy, Medical Faculty University of Duisburg‐Essen Essen Germany

**Keywords:** cerebellar brain inhibition, cerebellar tDCS, cerebellar–cerebral connectivity, transcranial magnetic stimulation

## Abstract

**Introduction:**

Cerebellar brain inhibition (CBI) is a way to quantify the cerebellar influence on the motor cortex in humans. Studies suggest that the orientation of the transcranial magnetic stimulation (TMS) coil influences which motor networks are activated.

This study investigated the influence of cerebellar transcranial direct current stimulation (ctDCS) on CBI as a function of coil orientation (anterior–posterior [AP] vs. posterior–anterior [PA]). An interstimulus interval (ISI) of 7 ms (CBI‐AP‐7) was used for AP orientation and 5 ms (CBI‐PA‐5) for PA orientation.

**Methods:**

Young and healthy participants received anodal, cathodal, or sham ctDCS treatment for 15 min on three different days. On each day, CBI was determined for both coil positions immediately after and 30, 60, and 120 min after ctDCS application.

**Results:**

For CBI‐PA‐5, no significant ctDCS effect was detected. For CBI‐AP‐7, there was an increase in CBI by anodal ctDCS and a decrease in CBI by cathodal ctDCS, although the latter did not reach statistical significance.

**Conclusion:**

Findings provide further support that different cerebello–cerebral motor networks may be activated in CBI‐AP‐7 and CBI‐PA‐5, with only CBI‐AP‐7 being significantly affected by ctDCS. CBI‐AP‐7 may be a more sensitive tool for investigating CBI effects than the CBI‐PA‐5 procedure, which is most commonly used.

## Graphical abstract

The impact of cerebellar direct current stimulation (ctDCS) on cerebellar brain inhibition (CBI) as a function of TMS coil orientation was investigated. Anodal ctDCS led to an increase in CBI for the anterior‐posterior (AP) orientation only. These results implicate, that different motor networks may be activated depending on the coil orientation and that CBI‐AP‐7 (CBI measured with AP position of the TMS coil and an interstimulus interval of 7 ms) may be a more sensitive tool for investigating CBI effects.

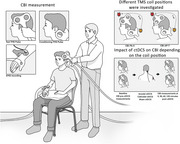

## 1 Introduction

Cerebellar brain inhibition (CBI) is a noninvasive transcranial magnetic stimulation (TMS) technique to test cerebello–cerebral interactions in humans (Ugawa et al. 1991). Here, a conditioning TMS stimulus is delivered toward the cerebellum before the application of a test stimulus over the contralateral primary motor cortex. The conditioning stimulus (CS) leads to a reduction in the amplitude of the motor evoked potential (MEP). Purkinje cells in the cerebellar cortex are thought to be activated by the cerebellar TMS pulse, thereby exerting an inhibitory influence on the deep cerebellar nuclei, which leads to reduced excitation of the cerebello‐thalamic‐M1 circuit (Hallett et al. 2017; Pinto and Chen 2001; Schlerf et al. 2015; Ugawa, Terao et al. 1995). The term CBI thus describes the reduction of the MEP by the preceding CS. The lower the measured (conditioned) MEP, the greater the CBI.

CBI can be influenced by neuroplastic processes induced by learning paradigms and by noninvasive cerebellar stimulation such as transcranial direct current stimulation (tDCS) or TMS. tDCS is a noninvasive technique that uses weak direct current application to modify cortical excitability and neuronal activity (Grimaldi et al. 2014). Numerous tDCS studies have shown promising, albeit sometimes contradictory, results (Galea et al. 2009; Jayaram et al. 2012). In a seminal paper, Galea et al. (2009) found a polarity‐specific effect of cerebellar tDCS on CBI, with cathodal tDCS leading to a clear and intensity‐dependent decrease in CBI and anodal tDCS to an increase. An increase in excitability at the level of the Purkinje cells due to anodal ctDCS is assumed to be the underlying mechanism (Ferrucci and Priori 2014; Grimaldi et al. 2014; Nitsche and Paulus 2000). Subsequent studies were able to replicate these findings only in part (Batsikadze et al. 2019). The rapidly growing field of continuous theta burst transcranial magnetic stimulation (cTBS) studies must also be mentioned at this point. Strzalkowski et al. (2019), for example, were able to show a significant reduction of CBI in healthy adults using two different cTBS protocols. In the study by Popa et al. (2010), a clear reduction in CBI was also demonstrated by both low‐frequency inhibitory repetitive transcranial magnetic stimulation (rTMS) over both cerebellar hemispheres and by cTBS of the right cerebellar hemisphere.

Not only the different stimulation techniques mentioned above, but also the parameters used for this seem to have an influence on the CBI. The orientation of the electric field induced by the coil position also seems to have a modulatory effect on CBI. Spampinato et al. (2020) investigated the effect of different interstimulus intervals (ISIs) on CBI in both anterior–posterior (AP) and posterior–anterior (PA) orientation of the TMS coil applying the test pulse. In a PA aligned coil, CBI was found to be greatest at an ISI of 5 ms, whereas for an AP aligned coil, maximal CBI was obtained at an ISI of 7 ms (Spampinato et al. 2020). Different cerebral motor networks (being connected with the cerebellum) may be activated by PA and AP currents. The research group investigated this in different experiments, initially regarding CBI as a function of coil position. Modulation by paired associative stimulation (PAS) and two different motor learning tasks showed different effects on CBI depending on the coil position. This assumption is supported by anatomical data showing a connection of different dentate parts to motor arm and leg areas on the one hand and premotor and other frontal areas on the other (Dum and Strick 2003). Beyond that, it seems plausible that different ISIs (to achieve maximum CBI) correspond to different interneuronal conduction times. A modeling study by Aberra et al. (2020) suggests that different layers of the neocortex are reached by the different coil positions. PA currents may stimulate pyramidal cells of the cortex layer V, while AP currents might reach more apical areas of the gyrus that receive projections from premotor areas (Aberra et al. 2020). In the AP coil position with the longer ISI of 7 ms, primarily cerebello‐premotor connections might be important. The longer ISI might reflect the additional interconnection in premotor areas. Likewise, Hamada et al. (2014) examined the effect of ctDCS on PAS with peripheral stimulation of the median nerve combined with a test stimulus over M1 with two different coil positions (AP vs. PA). A 15‐min session of ctDCS was shown to exert an influence on AP mediated inputs but not on PA inputs (Hamada et al. 2014). Together, these earlier studies provide evidence for the existence of different cerebral motor networks that may be relevant to the above observations in CBI but at the same time many questions remain unanswered. In the present study we investigated the effect of ctDCS on CBI depending on coil position (AP vs. PA orientation). Based on the findings by Spampinato et al. (2020) and Hamada et al. (2014) we expected more prominent ctDCS effects on CBI using the AP orientation.

## 2 Methods

### 2.1 Participants

A total of 21 young, healthy participants were included in the study after oral and written informed consent, of whom 16 were included in the final data analysis. Four participants had to be excluded: two could not complete the experiment due to intolerable side effects (headache), and two participants were excluded due to increased brainstem motor threshold (BST) (for details, see below). Another participant had to be excluded because he was identified as an extreme outlier, probably due to a measurement error.

The study received approval from the local ethics committee of the Medical Faculty of the University of Duisburg‐Essen (15‐6324‐BO) and was conducted in accordance with the Declaration of Helsinki.

Inclusion criteria for participation were an age between 20 and 30 years and right‐handedness (based on the Edinburgh Handedness Inventory; Oldfield 1971). Exclusion criteria were contraindications for the performance of TMS or tDCS (e.g., metallic implants), neurological or relevant internal diseases, the intake of centrally acting drugs, and the presence of pregnancy. To ensure this, all participants received a detailed neurological examination by an experienced physician from the Department of Neurology (AN) before the start of the experiment.

### 2.2 Procedure of the Experiment

The experiment consisted of three ctDCS sessions on three different days. Participants received either anodal, cathodal, or sham ctDCS. The order of the treatment conditions was randomly assigned. Both the participants and the investigator were blinded to the condition of stimulation. A minimum interval of 1 week was maintained between sessions.

**FIGURE 1:**
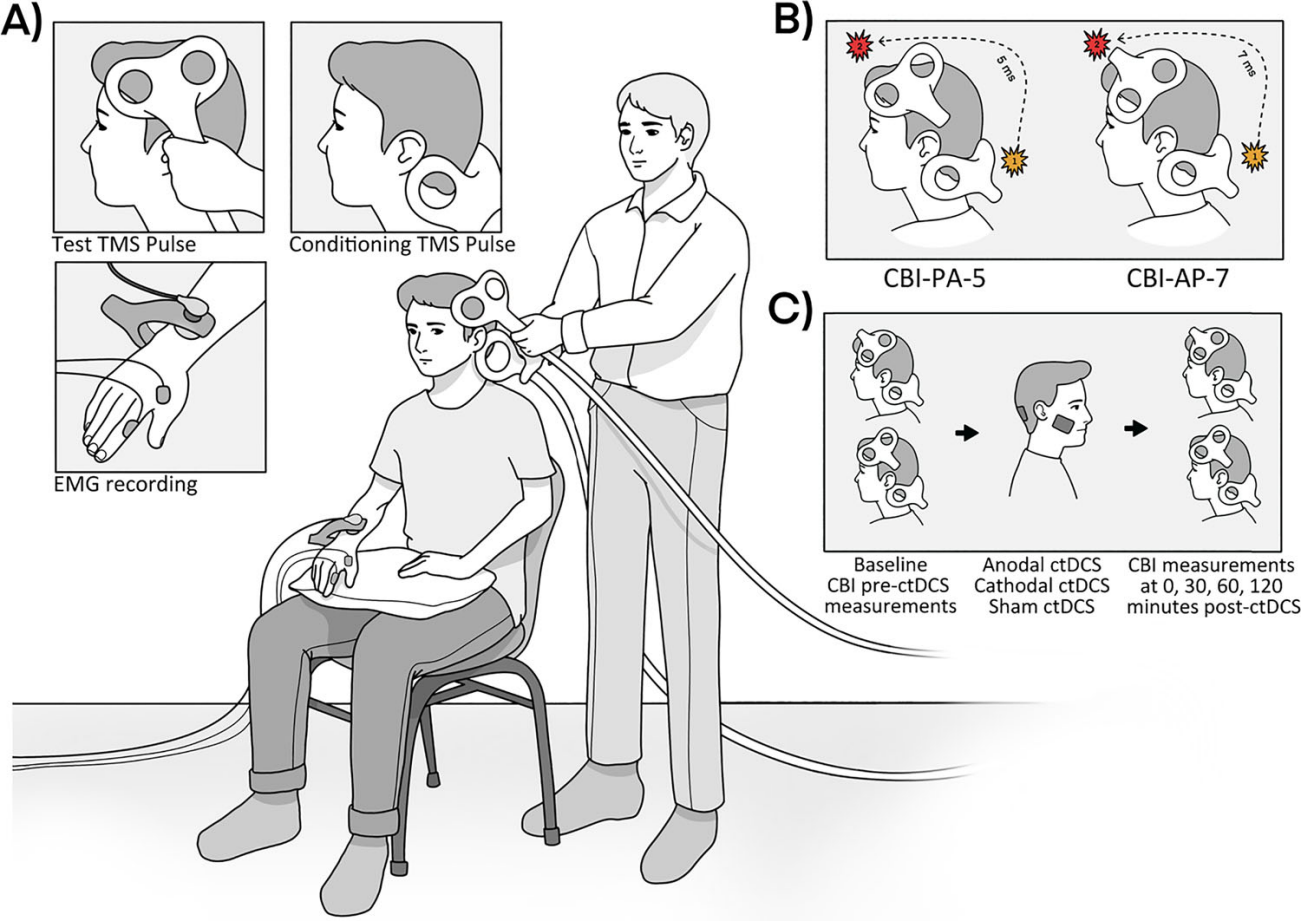
Experimental setup. (A) CBI measurement. A conditioning TMS stimulus is delivered toward the ipsilateral cerebellum before the application of a test stimulus over the contralateral primary motor cortex, which leads to a reduction in the amplitude of the motor evoked potential (MEP). (B) Coil orientation. CBI measurements were taken at both the AP coil orientation (with an ISI of 7 ms) and the PA coil orientation (with an ISI of 5 ms). (C) Cerebellar tDCS (ctDCS). After the baseline measurement, the participants received either anodal, cathodal, or sham ctDCS for 15 min, after which post‐ctDCS CBI measurements were recorded at 0, 30, 60, and 120 min post‐ctDCS.

Each session began with the measurement of the pre‐interventional (= pre‐ctDCS) CBI for both coil orientations (Figure 1C). In the next step, participants received cerebellar tDCS (ctDCS) (either anodal, cathodal, or sham) for 15 min. Immediately afterwards and after 30, 60, and 120 min (hereafter referred to as *T*_0_, *T*_30_, *T*_60_, and *T*_120_), the post‐interventional (= post‐ctDCS) CBI was determined for both coil orientations (Figure 1C).

### 2.3 TMS and CBI

The setup of the experiment is shown in Figure 1. A figure‐of‐eight magnetic coil (70 mm diameter; Magstim 200 magnetic stimulator, Magstim, UK) was used for the test stimulus. Single‐pulse TMS was applied over the left primary motor cortex to determine the motor threshold (electromyography, EMG of the right first dorsal interosseous [FDI] muscle). The “hot spot” was determined for both coil positions and used accordingly for the experiment. The participants received an individual cap on which the hotspot was marked. The lowest TMS intensity to elicit an MEP of an amplitude of 1 mV (SI1mV) was measured for both coil positions. “Hot spot” and SI1mV were maintained for the duration of the experiment (before and after ctDCS). The PA coil position was defined such that the coil stem was rotated 45° to the left from the sagittal suture (Figure 1B). The AP position was realized in such a way that the coil was rotated exactly 180° from the described (PA) position (Figure 1B).

A double‐cone coil (Magstim, UK) was used for the CS, which was applied over the right cerebellar cortex (ipsilateral to the EMG measurement), that is, 3 cm lateral to the inion. The current direction of the coil was downward (Figure 1A). At first, the motor threshold (BST) (Galea et al. 2009; Ugawa, Uesaka et al. 1995) was determined, limited to a maximum of 75% of the maximum stimulator output (MSO).

To determine the CBI, an intensity 5% lower than that of the BST was chosen as the CS. SI1mV was applied over the previously determined hotspot of the motor cortex (target muscle FDA) as the test stimulus. The ISI was 5 ms for the CBI‐PA and 7 ms for the CBI‐AP (Spampinato et al. 2020). During the study, all participants received blocks of 22 stimuli, of which 11 were elicited in randomized order as paired CS‐TS stimuli and 11 as unpaired TS stimuli. The time interval between paired/unpaired stimuli was randomized to 4 ± 0.4 s. Because the CBI for the AP and PA coil positions were determined for each measurement, there were thus 44 stimulations for each of the five measurement time points, that is, before application of ctDCS, immediately afterward, and after 30, 60, and 120 min, respectively (referred to as *T*_b_ = before ctDCS application, *T*_0_, *T*_30_, *T*_60_, and *T*_120_ for measurements immediately after stimulation and after 30, 60, and 120 min in the following, Figure 1C). The order of the applied coil positions (whether AP or PA first) was randomly assigned.

### 2.4 Cerebellar tDCS

ctDCS was applied using a Neuroconn DC stimulator at a current intensity of 2.0 mA (Batsikadze et al. 2019). Anodal and cathodal stimulation was each applied for 15 min (Hamada et al. 2014). In sham stimulation, current was ramped up for 10 s, remained at 2 mA for a duration of 30 s, after which current was ramped down again for 10 s. This is known to result in the same side effects (paresthesia) as in the case of verum stimulation but without leading to any observable neurophysiological effect (Ambrus et al. 2012; Dissanayaka et al. 2018). The cerebellar electrode (5 cm × 5 cm) was centered 3 cm lateral of the inion (Galea et al. 2009) to affect the right cerebellar cortex. The return electrode was placed over the right buccinator muscle (5 cm × 5 cm). Electrodes were fixed with Ten20 conductive gel (Neurodiagnostic Electrode Gel, Weaver and Company, USA) and tapes around the head. Both the investigator and the participants were blinded to the stimulation condition (anodal vs. cathodal vs. sham). This was realized in terms of the use of a custom‐built blinding box, which either interchanged or maintained stimulation polarities based on its setting.

### 2.5 Statistical Analysis

MEPs in which the muscle was not relaxed (clear EMG activity before the onset of the MEP above 10 mV) were manually excluded from the analysis. Outliers were defined as MEPs that were two standard deviations above—or below—the mean of each participant and were also excluded from the analysis. All MEP measurements were visualized manually in addition to the calculated assignment within the mean value ± two standard deviations. A maximum of 20% of the individual measurements per block and overall per test participant were excluded. If more measurements were outside this defined range, the entire data set was excluded from further analysis, which applied to one test participant as an extreme outlier. NuCursor software (Institute of Neurology, University College London, UK) was used to analyze EMG measurements.

CBI is defined as the quotient of the conditioned and unconditioned MEP amplitude. To compare the pre‐interventional and post‐interventional (after application of ctDCS) CBI, the difference with the pre‐interventional CBI was calculated for each post‐interventional time point (*T*_0_–*T*_120_) (ΔCBI *T*_0_–*T*_120_).

Data were analyzed with repeated measures ANOVA (RM‐ANOVA) separately for the raw and ΔCBI as dependent variables. The respective condition (anodal vs. cathodal vs. sham), CBI type (CBI‐PA‐5 vs. CBI‐AP‐7), and time (pre vs. post 0 vs. post 30 vs. post 60 vs. post 120) were used as within‐participant factors. As the baseline value was already included in the case of ΔCBI, only the points in time *T*_0_–*T*_120_ were considered for ΔCBI. In addition, post hoc RM‐ANOVA was performed separately on CBI‐PA‐5 and CBI‐AP‐7 data using condition (anodal vs. cathodal vs. sham) and time (*T*_0_–*T*_120_) as within‐participant factors.

RM‐ANOVA was also used to compare the stimulation intensities of motor thresholds and BST. The respective intensity (PA, AP, or BST) was used as a dependent variable, condition (anodal vs. cathodal vs. sham), and time (pre vs. post) were included as within‐participant factors. All analyses were conducted using SAS 3.81 (SAS Institute Inc., Cary, NC, USA). To address the negative bias in standard error and degrees of freedom calculations resulting from the small sample size, a Kenward–Roger adjustment was applied (Kenward and Roger 1997). Pairwise post hoc comparisons were performed using the least square means test when appropriate and adjusted for multiple comparisons using the Tukey–Krammer method. Partial eta squared (*η* ^2^) was used as a measure of effect size and calculated with the SAS macro developed by Tippey and Longnecker (2016).

## 3 Results

### 3.1 Effects of ctDCS on CBI

**FIGURE 2:**
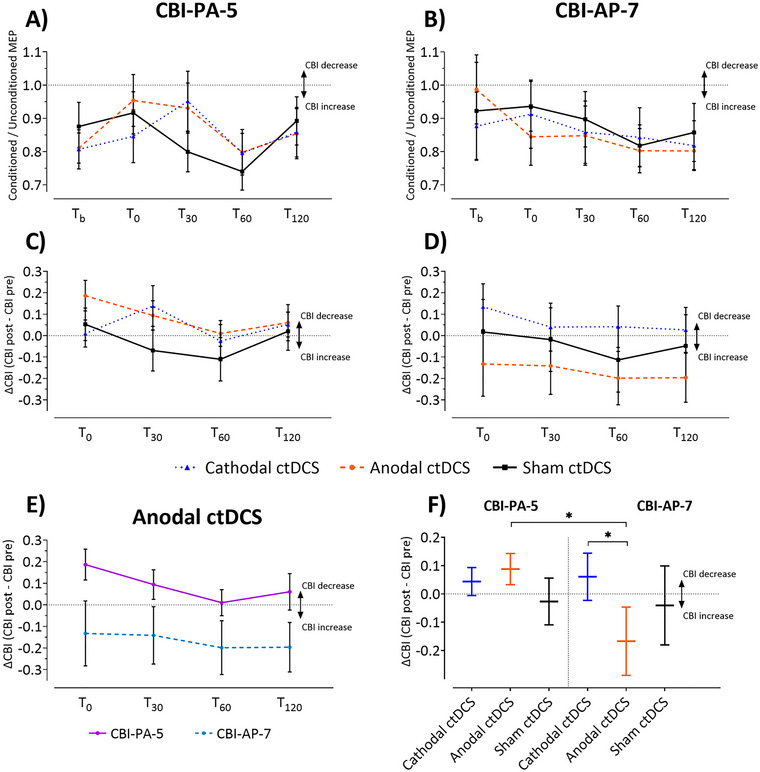
Raw and normalized data of the CBI measurements. (A, B) Raw CBI values. Shown are the results of the CBI measurements before (*T*_b_) and after (*T*_0_–*T*_120_) application of ctDCS for each of the three ctDCS conditions (anodal, cathodal, and sham). (C, D) Normalized data of the CBI measurements (in relation to the pre‐ctDCS CBI measurement) for each of the three ctDCS conditions (anodal, cathodal, and sham). ΔCBI [difference between the post‐ctDCS CBI (*T*_0_–*T*_120_) and the pre‐ctDCS CBI (*T*_b_)]. (A, C) The results of the CBI measurements for the PA coil position, and (B, D) the results for the AP coil position. (E) Effects of anodal ctDCS on CBI depending on the coil position. For the purpose of visualization, the effects of anodal ctDCS are shown integrated for both CBI‐PA‐5 and CBI‐AP‐7. (F) Collapsed data of CBI measurement (normalized data) over *T*_0_–*T*_120_ for both coil positions and different ctDCS conditions.

CBI before and after the application of ctDCS is shown in Figure 2. Raw data (CBI = quotient of unconditioned and conditioned MEP amplitude) are shown in Figure 2A,B with smaller values reflecting larger CBI effects. CBI measurements before ctDCS (Tb) and at the four time points after ctDCS (*T*_0_, *T*_30_, *T*_60_, and *T*_120_) for CBI‐PA‐5 and CBI‐AP‐7 are shown. To control for individual differences in baseline values (pre‐ctDCS; *T*_b_), data was normalized taking the initial value into account (Figure 2C,D) by calculating the difference between the pre‐ctDCS (*T*_b_) and post‐ctDCS CBI values at each time point (*T*_0_–*T*_120_) (Figure 2C,D).

It is important to note that in interpreting CBI, higher measured values (MEPs) correspond to a decrease in CBI (less inhibition), while lower MEPs correspond to an increase in CBI (more inhibition). We will henceforth describe the outcomes as either an increase or decrease in CBI to avoid confusion.

Regarding raw CBI values for the PA coil position, there is a decrease in CBI immediately after anodal stimulation (orange line in Figure 2A), which goes back to a level around baseline 120 min after stimulation (*T*_120_). For cathodal stimulation, a similar change can be observed for this coil position, but the decrease in CBI is only seen after 30 min and then also aligns with the baseline measure (blue line in Figure 2A). For sham stimulation, a minimal decrease in CBI, followed by an increase, is observable (black line in Figure 2A). For time point *T*_120_, all three arms show similar values close to the baseline.

For the AP coil position, a different picture emerges: Here, a clear increase in CBI can be observed after anodal stimulation (orange line in Figure 2B), which increases even further from time *T*_0_ to *T*_120_. After cathodal stimulation, on the other hand, there is a slight decrease of CBI at time *T*_30_, which subsequently increases again and remains at a level around the initial value at time *T*_120_ (blue line in Figure 2B). For sham stimulation, there is hardly any change from baseline at time points *T*_0_ and *T*_30_, and a slight increase in CBI at *T*_60_, which almost returns to baseline by *T*_120_ (black line in Figure 2B).

Main analysis is based on normalized values as the different baseline values would otherwise not allow an assessment of the dynamics. An overview of the statistical results is given in Table 1.

**TABLE 1:** Results of the repeated‐measures ANOVA.

Parameter	Factor	Num df, den df^a^	*F*	*p*	Partial *η* ^2^
Both coil positions					
CBI	Polarity Coil position Polarity × coil position Time Polarity × time Coil position × time Polarity × coil position × time	2, 435 1, 435 2, 435 4, 435 8, 435 4, 435 8, 435	0.10 0.11 0.65 1.90 0.43 0.60 0.88	0.904 0.743 0.524 0.110 0.905 0.662 0.536	0.0004 0.0002 0.0027 0.0157 0.0072 0.0050 0.0147
ΔCBI	Polarity Coil position Polarity × coil position Time Polarity × time Coil position × time Polarity × coil position × time	2, 345 1, 345 2, 345 4, 345 8, 345 4, 345 8, 345	2.01 4.01 4.21 1.21 0.14 0.12 0.28	0.136 **0.046** ^*^ **0.016** ^*^ 0.305 0.991 0.949 0.944	0.0009 0.0002 0.0078 0.0221 0.0053 0.0022 0.0106
Baseline raw CBI values					
CBI	Polarity Coil position Polarity × coil position	2, 75 2, 75 2, 75	0.54 1.14 0.72	0.586 0.290 0.489	0.0113 0.0118 0.0151
Conditioning and test pulse intensities					
PA Intensity	Polarity Time Polarity × time	2, 75 1, 75 2, 75	3.45 3.13 0.08	**0.037** ^*^ 0.081 0.927	0.0684 0.0319 0.0016
AP intensity	Polarity Time Polarity × time	2, 75 1, 75 2, 75	3.24 4.99 0.45	**0.045** ^*^ **0.028** ^*^ 0.639	0.0645 0.0499 0.0095
BST intensity^b^	Polarity	2, 75	2.50	0.089	0.0505

^a^ Degrees of freedom were adjusted using Kenward–Roger method (Kenward and Roger 1997).

^b^ The identical pre and post measurements in BST resulted in a non‐estimable effect for time or the polarity × time interaction.

* Significant effect at *p* < 0.05.

The major finding of the present study is a significant difference in coil position, indicated by a significant effect of coil position (*F*_1,345_ = 4.01, *p* = 0.046), and a significant polarity × coil position interaction [*F*_2,345_ = 4.21, *p* = 0.016) reflecting significant ctDCS effects depending on the coil position. Post hoc analysis of the polarity × coil position interaction revealed significantly different CBI in the cathodal compared to the anodal ctDCS condition measured in the AP coil position (Figure 2F, *p* = 0.023, least squares means test, Tukey–Krammer adjustment). In addition, a significant difference in CBI was observed when comparing the AP and PA coil positions post‐anodal ctDCS (Figure 2E, *p* = 0.007, least squares means test, Tukey–Krammer adjustment).

### 3.1 Test Pulses and BST Conditioning Pulses

**FIGURE 3:**
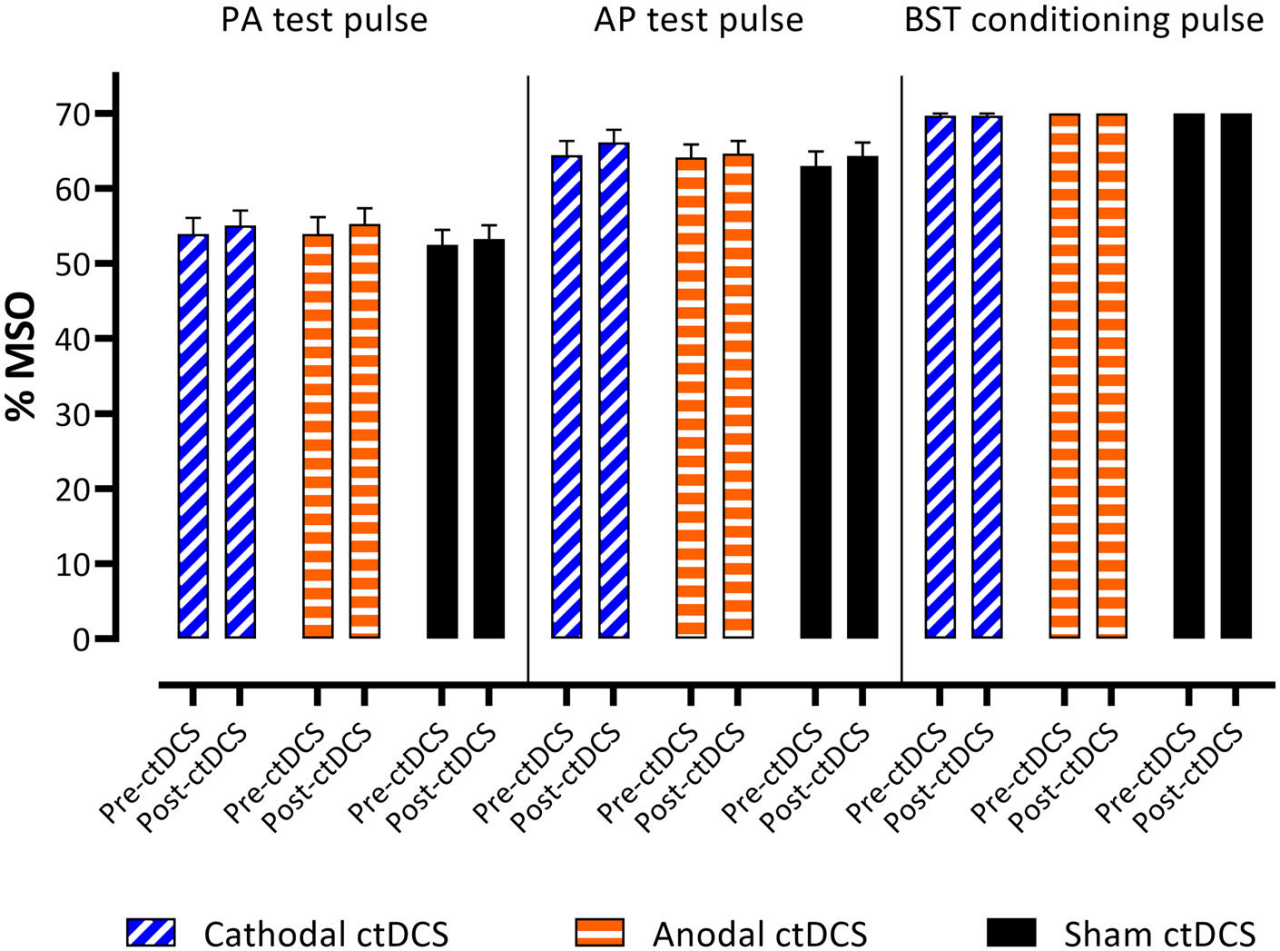
Mean intensities for the test pulse used for the PA and AP positions and the brainstem motor threshold (BST) before and after ctDCS administration are shown, revealing no significant differences in BST conditioning pulses but significantly higher post‐ctDCS test pulses compared to pre‐ctDCS test pulses. %MSO = % of maximal stimulator output; blue indicates the cathodal ctDCS session, red indicates the anodal ctDCS session, and green indicates the sham ctDCS session. Error bars depict SEM.

RM‐ANOVA revealed no statistically significant differences in BST intensities between three polarities (*p* = 0.089), pre‐ and post‐ctDCS BST values were identical (Table 2; Figure 3).

**TABLE 2:** Participant's characteristics and stimulation intensities.

		PA						AP						BST					
		Anodal ctDCS		Cathodal ctDCS		Sham ctDCS		Anodal ctDCS		Cathodal ctDCS		Sham ctDCS		Anodal ctDCS		Cathodal ctDCS		Sham ctDCS	
ID	Age/sex	Pre ctDCS	Post ctDCS	Pre ctDCS	Post ctDCS	Pre ctDCS	Post ctDCS	Pre ctDCS	Post ctDCS	Pre ctDCS	Post ctDCS	Pre ctDCS	Post ctDCS	Pre ctDCS	Post ctDCS	Pre ctDCS	Post ctDCS	Pre ctDCS	Post ctDCS
1	28/M	65	65	65	65	60	60	70	70	70	70	68	68	70	70	70	70	70	70
2	29/M	60	60	60	60	60	55	70	70	70	70	75	75	70	70	70	70	70	70
3	22/M	55	60	60	60	55	55	75	75	75	75	70	70	70	70	70	70	70	70
4	25/M	50	50	50	48	50	50	63	65	60	63	65	63	70	70	70	70	70	70
5	20/M	40	40	40	40	40	40	53	54	55	55	50	50	70	70	70	70	70	70
6	26/M	50	50	50	50	50	55	60	60	55	55	60	60	70	70	70	70	70	70
7	25/M	60	60	65	65	60	60	65	70	75	75	65	70	70	70	70	70	70	70
8	21/M	40	43	45	45	40	42	50	55	50	55	50	53	70	70	70	70	70	70
9	20/F	70	70	65	65	65	65	70	70	70	70	70	70	70	70	70	70	70	70
10	20/F	55	58	42	50	50	55	65	65	63	62	60	65	70	70	70	70	70	70
11	22/F	45	50	50	50	50	55	60	60	60	65	60	65	70	70	70	70	70	70
12	27/F	45	45	50	50	45	45	60	55	65	68	55	55	70	70	65	65	70	70
13	23/F	50	55	55	60	45	45	60	60	60	65	55	60	70	70	70	70	70	70
14	24/F	58	58	48	55	50	50	65	65	65	70	65	65	70	70	70	70	70	70
15	25/F	65	65	65	65	65	65	75	75	75	75	75	75	70	70	70	70	70	70
16	27/F	55	55	53	53	55	55	65	65	63	65	65	65	70	70	70	70	70	70

*Note*: Stimulations intensities in % of the maximum stimulator output (%MSO).

Abbreviations: AP, anterior–posterior coil position; BST, brainstem threshold; ctDCS, cerebellar transcranial direct current stimulation; f, female; m, male; PA, posterior–anterior coil position; y, years.

For the test pulse intensities, RM‐ANOVA revealed a significant effect of polarity (*F*_2,75_ = 3.24; *p* = 0.045) and time (*F*_1,75_ = 4.99; *p* = 0.028) for the AP position. The polarity × time interaction was not significant (*p* = 0.639; Table 1). Post hoc pairwise comparisons revealed significantly higher %MSO values in cathodal polarity compared to sham (*p* = 0.035, least squares means test, Tukey–Krammer adjustment); other comparisons (cathodal vs. anodal, anodal vs. sham) were not significant (both *p* ≥ 0.337).

For the PA intensity, RM ANOVA revealed a significant effect of polarity (*F*_2,75_ = 3.45; *p* = 0.037), but no significant differences in time or polarity × time interaction (both *p* ≥ 0.081; Table 2; Figure 3) were found. Post hoc pairwise comparisons revealed higher %MSO values in both cathodal and anodal conditions compared to sham; however these differences did not survive correction for multiple comparisons (both *p* ≥ 0.057, least squares means test, Tukey–Krammer adjustment).

Figure 3 shows higher test pulse values for the AP (compared to the PA) coil position. It is known that the TMS‐induced electric current that flows PA usually evokes highly synchronized corticospinal activity, whereas stimulation by AP evokes less synchronized and often slightly delayed corticospinal activity (Di Lazzaro et al. 2001). Different synaptic circuits are thought to be the underlying explanation (Di Lazzaro et al. 2001). Therefore, significantly higher intensities seem to be required for the AP position to trigger a corresponding MEP. Nevertheless, the overall differences are very small.

## 4 Discussion

The aim of the present study was to investigate the effects of TMS coil orientation when regarding the impact of anodal and cathodal ctDCS compared with sham ctDCS on CBI. Based on the assumption of two different cerebral motor networks targeted by CBI‐AP‐7 and CBI‐PA‐5, we hypothesized a differential effect of ctDCS on CBI depending on the coil orientation. In the study by Spampinato et al. (2020), the hypothesis was formulated that there are two different networks connecting the cerebellum and the motor cortex. The research group investigated this in different experiments, initially regarding CBI as a function of coil position. The finding that different ISIs were needed to generate maximum CBI depending on the coil position supports the assumption of two different interneuronal networks. It seems plausible that different ISIs (to achieve maximum CBI) correspond to different interneuronal conduction times.

For the measurement of CBI‐PA‐5, both after anodal and cathodal stimulation, a slight CBI decrease was shown, which statistically did not reach significance. Thus, the effects of CBI‐PA‐5 on CBI were small and independent of polarity. A different picture emerged for CBI‐AP‐7, where we saw an increase in CBI after anodal ctDCS. Therefore, anodal ctDCS for this coil position increased the influence of the cerebellum on M1 to a statistically significant extent. Unlike CBI‐PA‐5, we also saw a polarity‐dependent effect for CBI‐AP‐7, which became statistically significant. Cathodal ctDCS led to a decrease in the CBI—thus attenuating the cerebellar influence on M1.

Overall, a relevant influence of ctDCS on CBI was seen only for the AP coil position at an ISI of 7 ms. CBI‐AP‐7 appears to be more sensitive to the influence of ctDCS than CBI‐PA‐5. As yet, CBI‐PA‐5 has been most commonly used to assess CBI and to assess the effect of ctDCS in the literature (Batsikadze et al. 2019; Galea et al. 2009). Findings measuring the effect of ctDCS on CBI‐PA‐5 in the literature are mixed. The findings of a polarity‐specific effect of anodal and cathodal ctDCS on CBI reported in the seminal paper by Galea et al. (2009) have been replicated by later studies only in part. One paper from our group showed a significant increase in CBI after both anodal and cathodal stimulation (Batsikadze et al. 2019), thus an increase in CBI independent of the polarity of the stimulation. Other studies demonstrated opposite or no significant effects of ctDCS on CBI or showed effects only for one polarity (Bocci et al. 2015; Cantarero et al. 2015; Doeltgen et al. 2016; Macher et al. 2014; Panouilleres et al.; Pope and Miall 2012). To date, this inconsistency in the data remains to some extent unclear. Various explanations have been discussed. These range from low effect sizes and thus too small sample sizes in some studies to suboptimal and overall very heterogeneous stimulation parameters (Rampersad et al. 2014) to different interindividual responses to ctDCS (Labruna et al. 2019), which may depend on genetic factors (van der Vliet et al. 2018). Our findings suggest that CBI‐AP‐7 may show more robust effects of ctDCS, but this needs to be tested in the future. It must be mentioned at this point that CBI‐AP‐7 has not yet been studied as comprehensively as the conventional CBI‐PA‐5. Further research is required to confirm the results mentioned, to check them for robustness, and to work out specific characteristics. Yet, a more frequent use of CBI‐AP‐7 in future studies could possibly reveal less inconsistent data in ctDCS studies.

As outlined in the introduction, CBI‐AP‐7 may target premotor areas (Spampinato et al. 2020). The cerebellum has well‐known connections with premotor areas. While the hand motor area is located in the anterior lobe of the cerebellum, functional connections between the cerebellum and premotor areas have been found in the posterior cerebellar lobe (Buckner et al. 2011; Kipping et al. 2013). Electric field modeling shows that the ctDCS effects are most prominent in the posterior cerebellar lobe (Figure 2) in Rauscher et al. (2020). Thus, ctDCS effects may also be more prominent in CBI‐AP‐7 because cerebellar areas connected with premotor but not motor cerebellar areas are primarily stimulated.

At this point, we are thus assuming a specific (or more specific) ctDCS effect on the areas that are particularly relevant for the aforementioned network and that is addressed by CBI‐AP‐7.

If the hypotheses formulated in this work regarding two different motor networks can be further confirmed, this could prove to be a more than promising starting point for further studies on the pathophysiology and identification of therapeutic targets in movement disorders. The assumption of two different underlying networks depending on motor learning tasks, as formulated in Spampinato et al. (2020), could play a key role in the development of rehabilitative measures.

### 4.1 Limitations

There is also a need to explain a few aspects of the CBI data. The differences in the baseline measurement pose a challenge for analysis and interpretation. In particular, the baseline value for CBI‐AP‐7 in the anodal stimulation group raises the question of why there was obviously no significant CBI at this point and what this means for understanding the measurement over time. In fact, the recorded CBI values always only reflect a snapshot of the cerebello–cerebral connections under the influence of the coil position used and, if applicable, stimulation. The scatter of both intra‐ and inter‐individual measurements is a major problem that limits the informative value of the present study. In future studies, this problem should be addressed by larger case numbers and repeated measurements and averaging. The change in the measured CBI under ctDCS, which was measured under the same conditions, therefore appears to be primarily relevant at this point.

The observations regarding CBI‐PA‐5 must also be seen under the limitations mentioned above. These are very small and statistically insignificant effects. It can be critically questioned whether the CBI decrease in the anodal CBI‐PA‐5 group is really a genuine effect that could be replicated with a larger number of cases.

In our view, this appears equally relevant for the next limitation, concerning the use of delta measurement values. Due to inter‐individual differences and the resulting different baseline values, it is difficult to assess the raw data in the present data set, so we have chosen to present delta values primarily for reasons of clarity. The influence of ctDCS on CBI at different coil positions, and thus the research question of the present study, measured under the same condition in each case, is not changed by this.

Another restriction is that in the present study a single conditioning intensity was used, which could explain the nonsignificant findings in the CBI‐PA‐5 condition, as in previous work CBI‐PA modulation due to ctDCS was only observed at lower conditioning intensities (Galea et al. 2009). Thus, a ceiling effect might explain the lack of significant CBI‐PA‐5 modulation by ctDCS.

Finally, we chose ∼4 s as the inter‐CBI stimulation interval to ensure comparability with a previous study of our lab (Batsikadze et al. 2019). There is currently no clear agreement on the optimum interval. Carry‐over effects due to this relatively short interval cannot be ruled out.

## 5 Conclusion

In the present study, different ctDCS effects were demonstrated depending on the coil position. A significant effect of ctDCS was demonstrated only for CBI‐AP‐7. These findings support previous assumptions that different cerebello–cerebral connections are activated depending on the TMS test coil orientation in CBI paradigms and verify considerations in the work of Spampinato et al. (2020). This work thus not only provides a contribution to optimize the CBI tool for mapping ctDCS effects, as AP orientation of the TMS test coil may reveal more robust CBI effects, but also brings further evidence for the heterogeneity of cerebello–cerebral connections. Further studies with larger case numbers, which should in particular also take different age groups into account, are needed to confirm the present results. Last but not least, a more thorough examination of these issues in a larger collective could help to clarify the currently inconsistent data from ctDCS studies and also provide valuable insights into the characteristics of cerebello–cerebral networks, which could also form the basis for future therapeutic approaches.

## Author Contributions

**Katharina M. Steiner**: writing–original draft, supervision, methodology, formal analysis. **Seyed Ali Nicksirat**: data curation, formal analysis, investigation, methodology, writing–review and editing. **Andreas Thieme**: conceptualization, methodology, investigation. **Lara Müntefering**: data curation, project administration. **Thomas M. Ernst**: conceptualization, supervision, software. **Dagmar Timmann**: conceptualization, funding acquisition, writing–review and editing, methodology, project administration, resources, supervision, validation. **Giorgi Batsikadze**: conceptualization, supervision, writing–review and editing, formal analysis, methodology, validation, project administration.

## Ethics Statement

This study was performed in line with the principles of the Declaration of Helsinki. Approval was granted by the Ethics Committee of the Medical Faculty of the University Duisburg‐Essen.

## Consent

Oral and written informed consent was obtained from all individual participants included in the study.

## Conflicts of Interest

The authors declare no conflicts of interest.

### Peer Review

The peer review history for this article is available at https://publons.com/publon/10.1002/brb3.70364.

## Data Availability

The data that support the findings of this study are available from the corresponding author upon reasonable request.
